# Parkin Promotes Mitophagic Cell Death in Adult Hippocampal Neural Stem Cells Following Insulin Withdrawal

**DOI:** 10.3389/fnmol.2019.00046

**Published:** 2019-02-22

**Authors:** Hyunhee Park, Kyung Min Chung, Hyun-Kyu An, Ji-Eun Gim, Jihyun Hong, Hanwoong Woo, Bongki Cho, Cheil Moon, Seong-Woon Yu

**Affiliations:** ^1^Department of Brain and Cognitive Sciences, Daegu Gyeongbuk Institute of Science and Technology, Daegu, South Korea; ^2^Neurometabolomics Research Center, Daegu Gyeongbuk Institute of Science and Technology, Daegu, South Korea

**Keywords:** autophagy-dependent cell death, c-Jun, hippocampal neural stem cells, mitophagy, Parkin

## Abstract

Regulated cell death (RCD) plays a fundamental role in human health and disease. Apoptosis is the best-studied mode of RCD, but the importance of other modes has recently been gaining attention. We have previously demonstrated that adult rat hippocampal neural stem (HCN) cells undergo autophagy-dependent cell death (ADCD) following insulin withdrawal. Here, we show that Parkin mediates mitophagy and ADCD in insulin-deprived HCN cells. Insulin withdrawal increased the amount of depolarized mitochondria and their colocalization with autophagosomes. Insulin withdrawal also upregulated both mRNA and protein levels of Parkin, gene knockout of which prevented mitophagy and ADCD. c-Jun is a transcriptional repressor of Parkin and is degraded by the proteasome following insulin withdrawal. In insulin-deprived HCN cells, Parkin is required for Ca^2+^ accumulation and depolarization of mitochondria at the early stages of mitophagy as well as for recognition and removal of depolarized mitochondria at later stages. In contrast to the pro-death role of Parkin during mitophagy, Parkin deletion rendered HCN cells susceptible to apoptosis, revealing distinct roles of Parkin depending on different modes of RCD. Taken together, these results indicate that Parkin is required for the induction of ADCD accompanying mitochondrial dysfunction in HCN cells following insulin withdrawal. Since impaired insulin signaling is implicated in hippocampal deficits in various neurodegenerative diseases and psychological disorders, these findings may help to understand the mechanisms underlying death of neural stem cells and develop novel therapeutic strategies aiming to improve neurogenesis and survival of neural stem cells.

## Introduction

Regulated cell death (RCD) is an evolutionarily conserved process and is tightly controlled by various intracellular signals and extracellular cues (Danial and Korsmeyer, 2004; Galluzzi et al., 2018). RCD is essential for normal development and maintenance of tissue homeostasis (Ellis et al., 1991; Coucouvanis and Martin, 1995). Therefore, dysregulation of RCD underlies a variety of human diseases, such as cancer, neurodegeneration, and autoimmunity RCD is currently categorized into 12 distinct cell death subroutines, with intrinsic and extrinsic apoptosis, necroptosis, and autophagy-dependent cell death (ADCD) as main subroutines based on morphological and biochemical criteria (Galluzzi et al., 2018). Cell shrinkage, membrane blebbing, nuclear DNA fragmentation, chromatin condensation, and activation of caspases are hallmarks of apoptosis (Galluzzi et al., 2007). Morphologically, necrosis is characterized by a rapid collapse of the plasma membrane and uncontrolled release of cytoplasmic components (Rello et al., 2005). Recent progress in the understanding of the regulatory mechanisms of necrosis suggests its regulatable nature, and has led to the coining of the term “necroptosis” (Galluzzi and Kroemer, 2008; Moriwaki et al., 2016).

Autophagy (“*self-eating*” in Greek) is a lysosome-dependent catabolic process characterized morphologically by an increased formation of autophagic vesicles (autophagosomes and autolysosomes) (Yang and Klionsky, 2010). Autophagy is essential for turnover and removal of dysfunctional or toxic intracellular components (Shintani and Klionsky, 2004). Autophagy can also recycle obsolete cellular constituents (Mizushima and Komatsu, 2011). Thereby, autophagy generally has protective and adaptive functions in response to cellular stress. Autophagy is mediated by the autophagy-related (Atg) genes (Klionsky et al., 2011). It begins by phagophore formation and elongation to generate double-membraned autophagosomes. For this process, microtubule-associated proteins 1A/1B light chain 3B (LC3, the mammalian homologue of the yeast Atg8 protein) is cleaved by Atg4 to produce LC3-I, which is a cytosolic form of LC3 (Satoo et al., 2009). When autophagosome formation is triggered, LC3-I is conjugated to phosphatidylethanolamine to form LC3-II, which is then recruited to the expanding phagophore membranes to facilitate autophagosome formation (Ichimura et al., 2000). Autophagosomes then mature into autolysosomes through fusion with the lysosomes for degradation and recycling of the autophagy cargos. An increase in the amount of LC3-II or in the number of LC3 puncta in autophagic vesicles can serve as an indicator of increased autophagy flux (Klionsky et al., 2016). p62 (sequestosome 1/SQSTM1) is a ubiquitin-binding autophagy adaptor protein, which becomes incorporated into the autophagosomes together with the delivered cargos for autophagic degradation (Pankiv et al., 2007). Thus, the degradation of p62 is regarded as another index of autophagy flux (Ichimura et al., 2008).

Contrary to the general notion of autophagy as a survival mechanism, there are reports that excessive or prolonged autophagy can induce cell death (Tsujimoto and Shimizu, 2005). Together with frequent observation of autophagy in cells undergoing RCD, these findings led to the concept of ADCD. ADCD, previously also known as autophagic cell death, was first proposed based on widespread morphological association of autophagy with cell death, but without implication of the causative role of autophagy in cell death (Tsujimoto and Shimizu, 2005). Therefore, it is still contentious whether autophagy plays a causative role in cell death, especially in mammals. Taking this controversy into account, ADCD is suggested as a type of RCD that requires autophagic machinery without participation of other cell death pathways (Galluzzi et al., 2018).

Insulin/insulin-like growth factor (IGF) family proteins are vital extracellular cues for the differentiation and survival of neural stem cells (NSCs) in the hippocampus (Åberg et al., 2000; Lichtenwalner et al., 2001). As such, adult hippocampal NSCs, abbreviated as HCN cells following the original description of their isolation, depend on insulin/IGF for survival in *in vitro* culture (Palmer et al., 1997). Interestingly, we found that insulin-deprived HCN cells undergo ADCD rather than apoptosis despite their intact apoptotic capability (Yu et al., 2008; Baek et al., 2009). Further study revealed that glycogen synthase kinase-3β (GSK3-3β) mediates ADCD in HCN cells (Yu et al., 2008; Baek et al., 2009; Ha et al., 2015). Pharmacological or genetic inactivation of GSK-3β decreased ADCD, while over-expression of the wild-type (WT) or constitutively active form of GSK-3β facilitated ADCD without apoptosis induction (Ha et al., 2015). Because a rise in the intracellular Ca^2+^ level is known to trigger autophagy (Høyer-Hansen et al., 2007), we next focused on the regulation of ADCD by Ca^2+^. In insulin-deprived HCN cells, intracellular Ca^2+^ level increases, mainly owing to its release from the endoplasmic reticulum (ER) mediated by the type 3 ryanodine receptor (RyR3) (Chung et al., 2016). RyR3-mediated increase in cytosolic Ca^2+^ activates AMP-activated protein kinase (AMPK), which leads to a novel phosphorylation of p62 and promotes mitophagy (Ha et al., 2017). Further study is needed to understand how mitophagy is regulated in insulin-deprived HCN cells.

Parkin is an E3 ubiquitin ligase, and more than 100 mutations in the Parkin-encoding *PARK2* gene are known to cause an autosomal recessive form of Parkinson’s disease (PD) (Dawson and Dawson, 2010). PD is characterized mainly by an array of motor impairments associated with progressive death of dopaminergic neurons in the substantia nigra pars compacta (Dauer and Przedborski, 2003). PD also affects a number of neuronal systems and causes various non-motor symptoms including neuropsychiatric manifestations and cognitive deficits such as early premotor dysfunction (Meissner et al., 2011). The relevance of Parkin in these cognitive symptoms is not well understood. An emerging role of Parkin is regulation of mitophagy (Narendra et al., 2008). Mitophagy is a particular mode of autophagy that removes damaged or dysfunctional mitochondria and thereby helps maintain mitochondrial quality and homeostasis (Lemasters, 2005). Since mitochondrial dysfunction is implicated in the pathogenesis of PD, the role of Parkin-mediated mitophagy in the regulation of mitochondrial function and dynamics has gained great attention.

Hippocampus is one of the neurogenic regions where new neurons are continuously generated throughout adulthood (Gould et al., 1997; Alvarez-Buylla and Lim, 2004). Adult hippocampal neurogenesis is implicated in hippocampal learning and memory, and is impaired in the aged or injured brain (Shors et al., 2001; Rodríguez et al., 2008). Given their highly dynamic nature and differentiation potential, NSCs residing in the neurogenic niches must be under tight control in terms of metabolism, mitochondrial homeostasis, and autophagy level. Of relevance to this notion, a recent report on the characteristics of mt-Keima mice, an *in vivo* model of mitophagy, suggested high basal level of mitophagy in the dentate gyrus (DG) areas of the adult hippocampus (Sun et al., 2015). However, it has not been studied whether adult NSCs require Parkin activity for mitophagy.

In the present study, we investigated the role of Parkin in mitophagy in HCN cells; this investigation was prompted by its roles in other cell types and the high rate of on-going mitophagy in the DG. We demonstrate that Parkin is upregulated through degradation of its transcriptional repressor, c-Jun, following insulin withdrawal. Parkin is required for mitophagy and plays a pro-death role during ADCD of HCN cells. On the other hand, Parkin plays an anti-apoptotic role in response to well-known apoptotic stimuli. Our findings suggest distinct functions of Parkin in the regulation of RCD of HCN cells depending on the cellular context.

## Materials and Methods

### Reagents and Antibodies

Antibodies against Parkin (4211), cleaved caspase 3 (9664), poly(ADP-ribose) polymerase (PARP) (9542), c-Jun (9165), and voltage-dependent anion channel (VDAC) (4866), phospho-SAPK/JNK (Thr183/Tyr185) (9251), horseradish peroxidase (HRP)-linked anti-mouse IgG (7076) were purchased from Cell Signaling Technology (Danvers, MA, United States). Antibodies against p62 (P0067, Sigma-Aldrich, Saint Louis, MO, United States), LC3B (100-2220, Novus Biologicals, City of Centennial, CO, United States), HRP-conjugated β-actin (47778, Santa Cruz Biotechnology, Dallas, TX, United States) and goat anti-rabbit IgG (H+L) secondary antibody (31460, Thermo Fisher Scientific, Carlsbad, CA, United States), PTEN-induced putative kinase 1 (PINK1) (BC100-494, Novus Biologicals) and ubiquitin phosphorylated at S65 residue (p-Ub-S65) (ABS1513-I, EMD Millipore, Burlington, MA, United States) were purchased at the indicated companies. Bafilomycin A1 (Baf.A1, Sigma-Aldrich), carbonyl cyanide 3-chlorophenylhydrazone (CCCP, Sigma-Aldrich), staurosporine (STS, Cell Signaling Technology), and necrostatin-1 (Invitrogen, Carlsbad, CA, United States) were purchased from the indicated companies.

### Cell Culture

HCN cells were cultured as we previously reported (Chung et al., 2015, 2016). In brief, cells were grown in chemically composed serum free medium containing Dulbecco’s modified Eagle’s medium/F-12 (12400-024, Thermo Fisher Scientific) supplemented with our own-made N2 components, which we made by mixing individual components including 5 mg/l insulin (Sigma-Aldrich), 16 mg/l putrescine dihydrochloride (Sigma-Aldrich), 100 mg/l transferrin (Sigma-Aldrich), 30 nM sodium selenite (Sigma-Aldrich), and 20 nM progesterone (Sigma-Aldrich). Medium was adjusted to pH 7.2 after adding 1.27 g/l sodium biocarbonate (Sigma-Aldrich). Insulin was omitted to prepare I(−) medium. In this paper, I(+) and I(−) denote insulin-containing and insulin-deprived media, respectively.

### Cell Death Assay

HCN cells were seeded in a 96-well plate at a density of 1.0 × 10^5^ cells/ml. To assess cell death, cells were stained with Hoechst 33342 (Invitrogen) and propidium iodide (PI) (Sigma-Aldrich), and were imaged under a fluorescence microscope (Axiovert 40 CFL, Carl Zeiss, Oberkochen, Germany). We used DAPI filter for Hoechst stained cells, and Cy3 filter for PI positive cells. Images were obtained by AxioVision 4 module Multichannel software (Carl Zeiss) and were analyzed by Pixcavator student edition analysis software (Intelligent Perception Co.). The contrast value of the software was adjusted according to the brightness of the image, and total 7,000–8,000 cells were counted per condition and more than 3 experiments were performed. The cell death rates were calculated as follows: Cell death (%) = (PI positive cell number/Hoechst positive cell number) × 100.

### Caspase 3 Activity Assay

HCN cells were seeded onto 96-well white plates and caspase 3 activity was measured using a Caspase 3 activity assay kit (Promega, Madison, WI, United States) according to the manufacturer’s instructions. A freshly prepared Caspase 3 Glo reagent solution was added to cultured HCN cells, and luminescence was measured in a luminometer (SpectraMax L, Molecular Devices, San Jose, CA, United States) and were analyzed by SoftMax Pro Software (Molecular Devices). Luminescence values were normalized to protein concentration. We calculated the average value by analyzing duplets samples per condition, and performed a total 3 repeat experiment.

### Terminal Deoxynucleotidyl Transferase dUTP Nick End Labeling (TUNEL) Assay

Cells were fixed in 4% paraformaldehyde for 25 min at 4°C, washed twice in phosphate-buffered saline (PBS), 5 min each time. After permeabilization with 0.2% Triton X-100 in PBS for 5 min, the assay was performed using TUNEL System kit (G3250, Promega) according to the manufacture’s instruction. In brief, 100 μl of equilibration buffer was added to the cells for 5–10 min at room temperature. After that, 50 μl TdT reaction mix was added for 60 min at 37°C. Cells were immersed in 2 × SSC buffer to stop reaction. Hoechst was added to stain nucleus, and the cells were mounted and analyzed by under a confocal microscope (LSM 700, Carl Zeiss).

**Table 1:** Information of plasmids.

Plasmid name	Plasmid numbers	Characteristics	Reference
pmRFP-LC3	21075	Encoding a fusion of rat LC3 and mRFP	Kimura et al., 2007
pEGFP-LC3	21073	Encoding a fusion of rat LC3 and EGFP	Kabeya et al., 2000
ptfLC3	21074	Encoding a fusion of rat LC3 and mRFP and EGFP	Kimura et al., 2007
pMXs-puro GFP-DFCP1	38269	Encoding a fusion of *M. musculus* DFCP1 and EGFP	Itakura and Mizushima, 2010
pDsRed2-Mito	632421	Encoding a fusion of *Discosoma* sp. red fluorescent protein (DsRed2) and a mitochondrial targeting sequence of human cytochrome c oxidase subunit VIII (Mito).	
pCase12-mito	FP992	Encoding mitochondria-targeted fluorescent Ca^2+^ sensor Case12	Gorman, 1985

### Western Blotting

HCN cells were harvested and lysed on ice in radioimmunoprecipitation assay (RIPA) buffer (Thermo Fisher Scientific) with 1 × Halt Protease and Phosphatase inhibitor cocktail (Thermo Fisher Scientific), 1 mM phenylmethanesulfonylfluoride, 1 mM dithiothreitol. Lysates were centrifuged 12,000 ×*g* for 10 min and protein concentration was measured using a Pierce BCA protein assay kit (Thermo Fisher Scientific). Usually, 10–20 μg total protein was loaded per well. After electrophoresis, proteins were transferred to polyvinylidene fluoride membranes in a semidry electrophoretic transfer cell (Bio-Rad, Richmond, CA, United States). The membranes were blocked in blocking buffer consisting of 5% non-fat dry milk in Tris-buffered saline with 0.1% Tween 20 (TBST) for 1 h at room temperature and incubated overnight with diluted primary antibodies. Each primary antibody was diluted in 5% bovine serum Albumin (Thermo Fisher Scientific) or 5% skim milk in TBST. After 3 washes with TBST, membranes were incubated with suitable HRP-conjugated secondary antibodies (10,000 dilution) diluted in blocking buffer for 1 h. After 3 washes with TBST, proteins were detected using Super Signal West Pico PLUS Chemiluminescent Substrate (34580, Thermo Fisher Scientific). SRX 201A (Konica Minolta Medical Imaging, Wayne, NJ, United States) was used to develop films. Obtained images were analyzed using Image J software and each protein level was finally quantified after normalization via β-actin.

### Mitochondrial and Cytosolic Fractionation

Subcellular fractionation was performed according to the published protocol (Dimauro et al., 2012) with a slight modification. HCN cells were homogenized by vortexing for 15 min in lysis buffer (250 mM sucrose, 50 mM Tris-HCl, pH 7.4, 5 mM MgCl_2_, 1 × Halt protease and phosphatase inhibitor cocktail) and kept on ice for 30 min. The homogenate was centrifuged at 600 ×*g* for 15 min and the supernatant (S_0_) was further centrifuged at 11,000 ×*g* for 10 min to separate the supernatant (S_1_) and pellet (P_1_). An equal volume of cold 100% acetone was added to S_1_ and the samples were incubated overnight at –20°C to precipitate the proteins. After centrifugation at 12,000 ×*g* for 5 min, the pellet was resuspended in lysis buffer and used as the cytosolic fraction. P_1_, which contained the mitochondrial fraction, was resuspended in lysis buffer and centrifuged at 11,000 ×*g* for 10 min; the pellet (P_2_) was resuspended in extraction buffer (50 mM Tris-HCl pH 6.8, 1 mM EDTA, 0.5% Triton-X 100, and protease and phosphatase inhibitor cocktails) and sonicated with a Bioruptor KRB-01 (CosmoBio, Japan) on ice 3 times for 10 s with 30 s intervals. We used this fraction as the mitochondrial fraction.

### Plasmids and Transfection

Plasmids encoding RFP-LC3, EGFP-LC3, monomeric RFP-GFP tandem fluorescent LC3 (mRFP-GFP-LC3), and GFP-double FYVE domain–containing protein 1 (DFCP1) were purchased from Addgene (Cambridge, MA, United States). DsRed2-Mito was purchased from Takara Bio United States (Mountain View, CA, United States) and Case12-mito from Evrogen (Moscow, Russia). Cells were seeded in a 6-well plate 24 h prior to transfection. Transfection was conducted using Lipofectamine 2000 (Invitrogen) according to the manufacturer’s instructions. The Plasmids list is shown in Table 1.

### *Park2* and *Pink1* Knockout

Rat *Park2* single guide RNA (sgRNA), rat *Pink1* sgRNA, and Cas9 were designed by and purchased from Toolgen (Republic of Korea). The target sequence for *Park2* was 5′-ATCACTCGCAGCTGGTCAGCTGG-3′ and the target sequence for *Pink1* was 5′-CCTGACACCGGGCCCGGCTTGGG-3′. HCN cells were transfected with *Park2* sgRNA or *Pink1* sgRNA and Cas9 for 24 h and selected by hygromycin B (InvivoGen, San Diego, CA, United States).

### Quantitative Real-Time Reverse Transcription Polymerase Chain Reaction (qRT-PCR)

RNA was isolated from HCN cells using QIAzol lysis reagent (Qiagen, Germantown, MD, United States) and used to synthesize cDNA with an ImProm-II Reverse Transcriptase kit (Promega). A CFX96 Real-Time PCR detection system (Bio-Rad) and TOPreal qPCR 2X premix (Enzynomics, Republic of Korea) were used for real-time PCR with the following primers: rat *Park2* forward (5′-ATG ATA GTG TTT GTC AGG TT-3′) and reverse (5′-AGA CAA AAA AGC TGT GGT AG-3′); rat ring finger protein 19A (*Rnf19a/Dofrin*) forward (5′-ATC TCC AAT CGT CTG CTT CGT CTG-3′) and reverse (5′-CGT TCA GTG CATTCT GGA CAA CTG-3′); rat heme-oxidized IRP2 ubiquitin ligase-1 (*HOIL-1/Rbck1*) forward (5′-ATG GAC GAG AAGACC AAG AAA GCA-3′) and reverse (5′-GTT GAG TGA TGT GTT GCG GGC T-3′); β-actin *(Actb)* forward (5′-AGC CAT GTA CGT AGC CAT CC-3′) and reverse (5′-CTC TCA GCT GTG GTG GTG AA-3′).

### Flow Cytometry Analysis

Before harvesting, HCN cells were incubated with 100 nM MitoTracker Deep Red and 50 nM MitoTracker Green (Thermo Fisher Scientific) for 15 min. Harvested cells were washed in cold PBS twice and analyzed using a BD Accuri C6 flow cytometer (BD Biosciences, San Jose, CA, United States). MitoTracker Green- and MitoTracker Deep Red-stained cells were detected by FL1 and FL3 channel, respectively. Total 30,000 cells were counted in each condition. For negative control, non-stained cells and single stained cells were counted. Data were analyzed by BD Accuri C6 software.

### Immunocytochemistry and Quantification of Relative Fluorescence Intensity or Colocalization Coefficient

Cells were fixed in 4% paraformaldehyde or methanol for 5–10 min, washed in PBS twice, and mounted on a glass slide with mount solution (Dako, Carpinteria, CA, United States). Samples were examined under a confocal microscope (LSM 700, Carl Zeiss). Relative fluorescence intensities of TMRE, Case12-mito, p-Ub-S65 were analyzed using Image J software. The cytosol region excluding nucleus area was drawn with drawing tools and integrated density of this region was measured. The relative fluorescence intensity in each experimental condition was quantified based on the mean value of the I(+) condition.

Colocalization coefficient of mitochondria with LC3 or DFCP1 was analyzed using “colocalization” tool from Zen software (Carl Zeiss) and then quantified using colocalization coefficient following the guideline of the software.

### Promoter Activity Assay

pGL3 and pRL-TK vectors (Promega) contain modified coding regions for firefly luciferase and *Renilla* luciferase, respectively. We used firefly luciferase as a reporter for transcriptional activity of *Park2* promoter. *Renilla* luciferase, which was expressed constitutively, served as a control for normalization. Cells were seeded in a 12-well plate 24 h prior to transfection, and pGL3-basic vectors carrying a series of truncated forms of the rat *Park2* promoter region were co-transfected with pRL-TK using Lipofectamine 2000. After transfection for 12 h, the cells were collected in I(-) medium and re-seeded in a 24-well plate. Luciferase assay was conducted by using a Dual-Glo Luciferase Assay System (Promega) according to the manufacturer’s instructions. Freshly prepared Dual-Glo luciferase reagent solution was added to the cells and luciferase activity was measured in a white 96-well plate in a SpectraMax L luminometer. We measured and analyzed the data using “photon counting mode” of SoftMax Pro Software. We calculated the relative values of each result based on I(+).

### Statistics

Data from at least three independent experiments were expressed as the mean ± standard error (SEM). “*n* = ” represents a number of cells, unless otherwise stated. Statistical significance was determined using the paired *t*-test for two-group experiments. For experiments with three or more groups, comparisons were made using one-way analysis of variance and Tukey’s test. GraphPad Prism 5 (GraphPad Software, San Diego, CA, United States) was used to analyze the data. Differences were considered statistically significant for *p*-values < 0.05.

## Results

### Insulin Withdrawal Induces ADCD With Mitochondrial Alterations in HCN Cells

**FIGURE 1:**
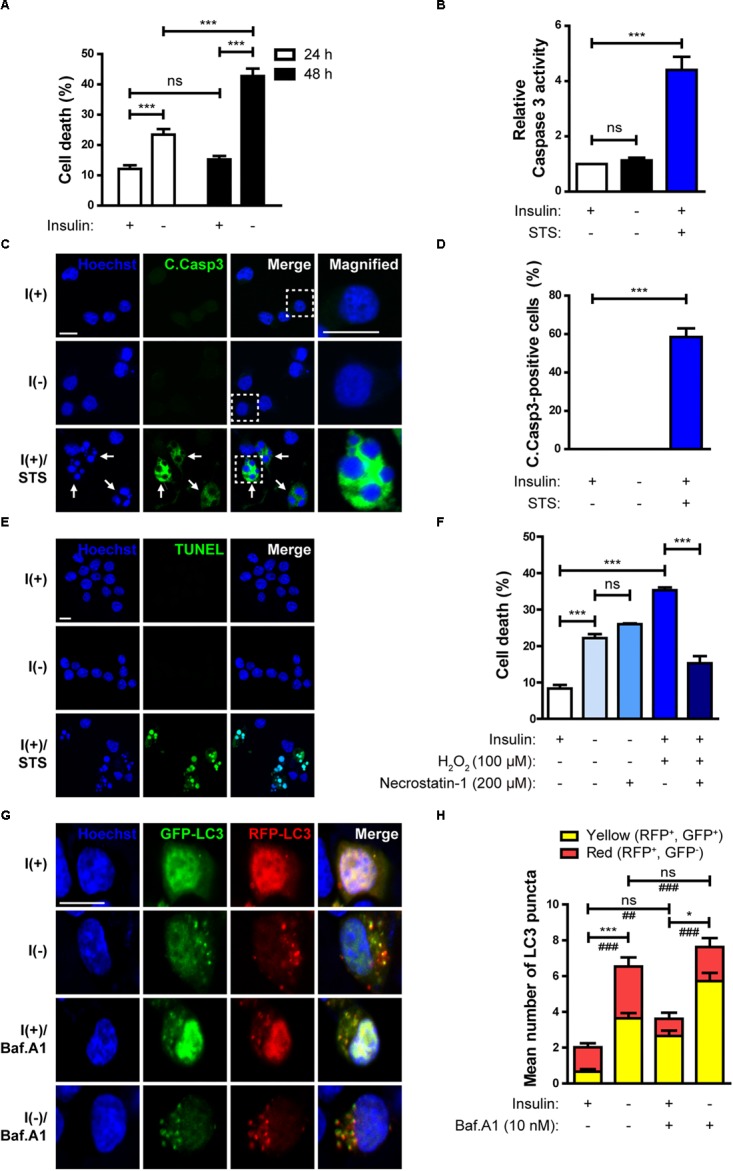
Insulin withdrawal induces autophagy-dependent cell death (ADCD) in adult hippocampal neural stem (HCN) cells. **(A)** Cell death rates in insulin-deprived HCN cells after insulin withdrawal for 24 and 48 h (*n* = 3). **(B)** Caspase 3 activity after insulin withdrawal for 24 h. HCN cells were treated with staurosporine (STS; 0.5 μM) for 3 h as a positive control of apoptosis (*n* = 4). **(C)** Immunocytochemical detection of cleaved caspase 3 (C.Casp3) in HCN cells after insulin withdrawal for 24 h. Cells were treated with STS (0.5 μM, 8 h) (*n* = 47 cells for I(+), 50 cells for I(-), 56 cells for I(+)/STS from 3 independent experiments). Arrows indicate fragmented nuclei in C.Casp3-positive cells. Scale bars, 10 μm. **(D)** Quantification of C.Casp3-positive cells from **(C)** (*n* = 47 cells for I(+), 50 cells for I(-), 56 cells for I(+)/STS from 3 independent experiments). **(E)** TUNEL assay in HCN cells after insulin withdrawal for 24 h. Cells were treated with STS (0.5 μM, 8 h) (*n* = 258 cells for I(+), *n* = 310 cells for I(-), *n* = 340 cells for I(+)/STS from 3 independent experiments). Scale bar, 10 μm. **(F)** Cell death rates in insulin-deprived HCN cells treated with necrostatin-1 after insulin withdrawal for 24 h (*n* = 3). **(G)** Assessment of autophagy flux by mRFP-GFP-LC3 puncta assay after insulin withdrawal for 24 h. Cells were treated with Baf.A1 (10 nM) for 1 h before harvesting. Scale bar, 10 μm. **(H)** Quantification of red and yellow LC3 puncta from **(G)** (*n* = 47 cells for I(+), 40 cells for I(+)/Baf.A1, 52 cells for I(-), 50 for I(-)/Baf.A1 from 3 independent experiments). ^∗^*p* < 0.05, ^∗∗∗^*p* < 0.001 for the red puncta; ^##^*p* < 0.01, ^###^*p* < 0.001 for the yellow puncta; ns, not significant.

HCN cells depend on insulin for their survival and proliferation (Palmer et al., 1997). Insulin withdrawal significantly increased cell death (Figure 1A). Consistent with our previous reports (Yu et al., 2008; Baek et al., 2009) on the non-apoptotic nature of insulin withdrawal-induced death of HCN cells, caspase 3 activity (Figure 1B) and the number of activated, cleaved caspase 3 (C.Casp3)-positive cells remained very low despite an increase in cell death, indicating no involvement of apoptosis (Figure 1C,D). On the other hand, a robust induction of caspase 3 activation by the well-known prototypical apoptosis inducer STS demonstrates normal apoptotic capability of HCN cells (Figure 1B–D). We also confirmed that DNA fragmentation, a marker of apoptosis, did not occur in insulin-deprived HCN cells (Figure 1E). 63% of total cells were TUNEL-positive by STS-treatments, but TUNEL-positive cells were not observed in either I(+) or I(-) condition. Also, ineffectiveness of pan caspase inhibitor Z-VAD against insulin withdrawal-induced death of HCN cells have been reported in our previous studies (Chung et al., 2015; Ha et al., 2015). These results support non-apoptotic nature of insulin withdrawal-induced HCN cell death. In addition, insulin-deprived HCN cells did not undergo necrosis. Necrostatin-1, an inhibitor of necroptosis, blocked cell death induced by H_2_O_2_ but did not prevent cell death induced by insulin withdrawal (Figure 1F). We also reported that suppression of autophagy by knockdown of Atg7 prevented HCN cell death following insulin withdrawal, indicating the causative role of autophagy in cell death (Yu et al., 2008). Characterization of cell death mode excluded apoptosis and necroptosis. Therefore, we presumed that PI/Hoechst-based cell death assay approximately measures total cell death under insulin withdrawal condition.

To measure autophagic flux, immunofluorescence assay using mRFP-GFP-LC3 was performed. The GFP, but not RFP signal is readily quenched in acidic conditions, such as within the autolysosomes (Kimura et al., 2007). As a result, autophagosomes and autolysosomes are observed as yellow (RFP^+^GFP^+^) and red (RFP^+^GFP^−^) puncta, respectively (Kimura et al., 2007). In insulin-deprived HCN cells, the number of both the yellow and red LC3 puncta increased, indicating an increase in autophagy flux in comparison with I(+) HCN cells (Figure 1G,H). In addition, when autophagosome maturation was blocked by treatment with Baf.A1, which inhibits fusion between autophagosomes and lysosomes, accumulation of yellow LC3 puncta in I(-) HCN cells was much higher than without Baf.A1 treatment, confirming a robust biogenesis of autophagic vesicles in I(-) HCN cells (Figure 1G,H).

Taken together, insulin withdrawal-induced cell death meets the following criteria: (i) increased autophagic flux is observed (this study; Yu et al., 2008; Baek et al., 2009); (ii) suppression of autophagy prevents cell death (Yu et al., 2008); and (iii) alternative cell death pathways such as apoptosis and necrosis are not involved (this study; Yu et al., 2008; Chung et al., 2015; Ha et al., 2015). Thus, from the current and our previous data we concluded that insulin-deprived HCN cells undergo ADCD.

**FIGURE 2:**
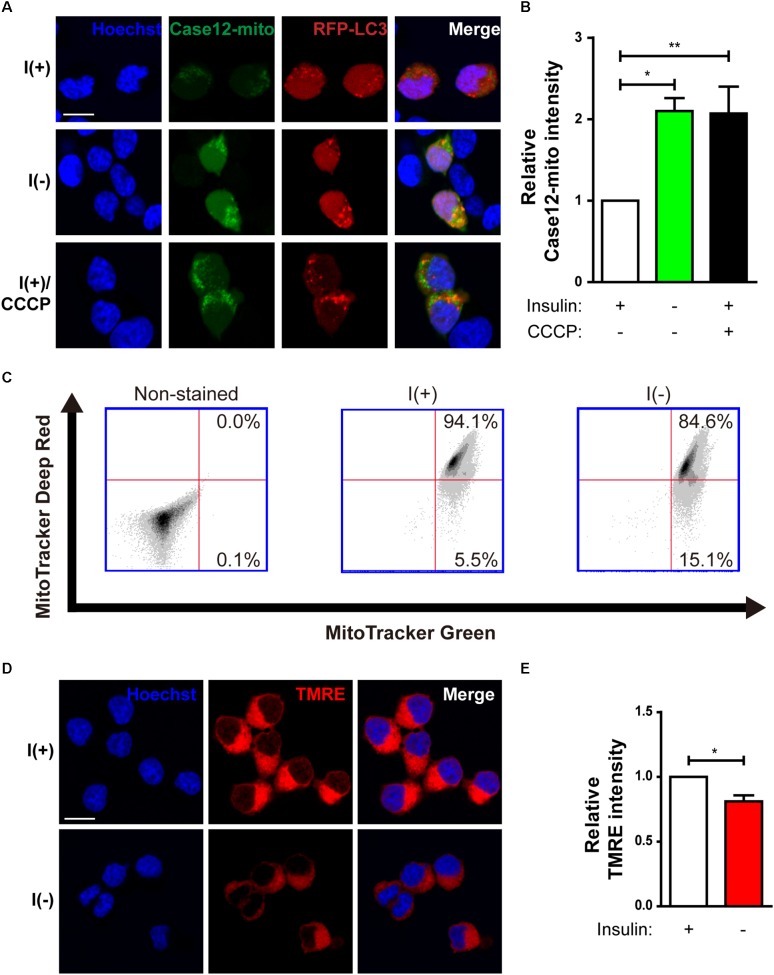
Changes in mitochondria of HCN cells after insulin withdrawal. **(A)** Assessment of mitochondrial Ca^2+^ levels using Case12-mito after insulin withdrawal for 24 h. Scale bar, 10 μm. **(B)** Quantification of fluorescence intensity of Case12-mito in HCN cells (*n* = 33 cells for I(+), 25 cells for I(-), 36 cells for I(+)/CCCP from 3 independent experiments). **(C)** FACS analysis of HCN cells stained with MitoTracker Green to determine the total number of mitochondria and MitoTracker Deep Red to determine the number of depolarized mitochondria after insulin withdrawal for 24 h. 30,000 cells were analyzed in each experiment. **(D)** Fluorescence imaging analysis of TMRE-stained HCN cells. Scale bar, 10 μm. **(E)** Quantification of fluorescence intensity of TMRE in HCN cells (*n* = 12 cells from 2 independent experiments). Relative TMRE intensity of fluorescence microscope image was measured by image J. ^∗^*p* < 0.05, ^∗∗^*p* < 0.01.

Our previous observation suggests that insulin withdrawal induces an increase in cytosolic Ca^2+^ that originates from the ER (Chung et al., 2016). We hypothesized that this increase may induce mitochondrial Ca^2+^ accumulation. To measure mitochondrial Ca^2+^ level, we co-transfected HCN cells with RFP-LC3 and Case12-mito, which is an indicator of mitochondrial Ca^2+^ level (Takeuchi et al., 2013). Co-transfection with RFP-LC3 and Case12-mito showed an increase in mitochondrial Ca^2+^ levels in autophagy-induced cells following insulin withdrawal (Figure 2A,B). Since elevated mitochondrial Ca^2+^ could cause mitochondrial depolarization (Baumgartner et al., 2009), we next examined mitochondrial depolarization by staining HCN cells with MitoTracker Deep Red or TMRE, which are sequestered in healthy, polarized mitochondria, but are excluded from depolarized mitochondria (Scaduto, R. C. Jr. and Grotyohann, 1999). Fluorescence-activated cell sorting (FACS) analysis of HCN cells co-stained with MitoTracker Green (to ensure the equal number of total mitochondria) and MitoTracker Deep Red (for measurement of mitochondria membrane potential) revealed a reduced number of MitoTracker Deep Red–positive cells, indicating an increase in mitochondrial depolarization following insulin withdrawal (Figure 2C). This result was confirmed by fluorescence microscopy, which showed lower staining intensity of TMRE in I(−) HCN cells than in I(+) HCN cells (Figure 2D,E).

**FIGURE 3:**
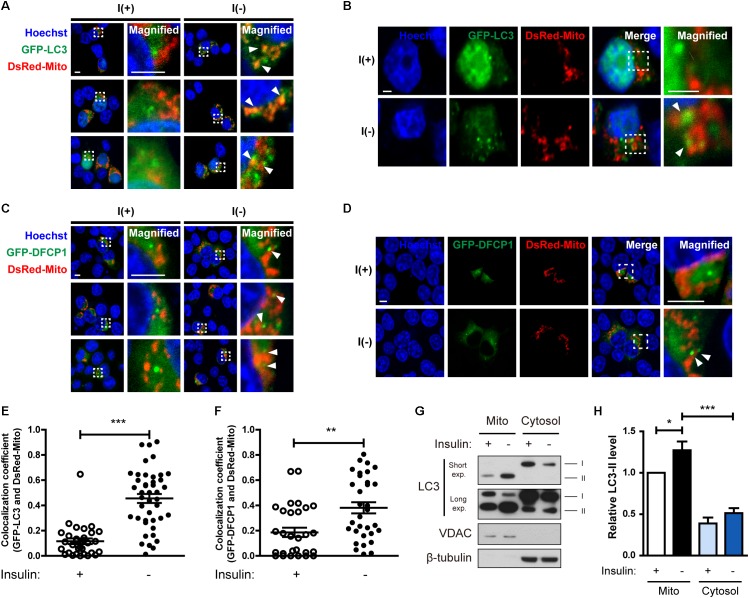
Insulin-deprived HCN cells undergo excessive mitophagy. **(A,B)** Colocalization of GFP-LC3 and DsRed-Mito (arrowheads) after insulin withdrawal for 24 h. Scale bars, 5 μm for **(A)**, 2 μm for **(B)**. **(C,D)** Immunofluorescence of GFP-DFCP1 and DsRed-Mito after insulin withdrawal for 24 h. Arrowheads indicate colocalization of GFP-DFCP1 and DsRed-Mito. Scale bars, 5 μm for **(C)**, 2 μm for **(D)**. **(E)** Quantification of colocalization of LC3 and mitochondria (*n* = 31 cells for I(+), 42 cells for I(-) from 3 independent experiments). **(F)** Quantification of colocalization of DFCP1 and mitochondria (*n* = 30 cells for I(+), 33 cells for I(-) from 3 independent experiments). **(G)** Western blotting analysis of mitochondrial translocation of LC3 after insulin withdrawal for 24 h. Mito, mitochondrial fraction; cytosol, cytosolic fraction. The blots shown are representative of four experiments with similar results. **(H)** Quantification of LC3-II levels (*n* = 4) from **(G)**. ^∗^*p* < 0.05, ^∗∗^*p* < 0.01, ^∗∗∗^*p* < 0.001.

**FIGURE 4:**
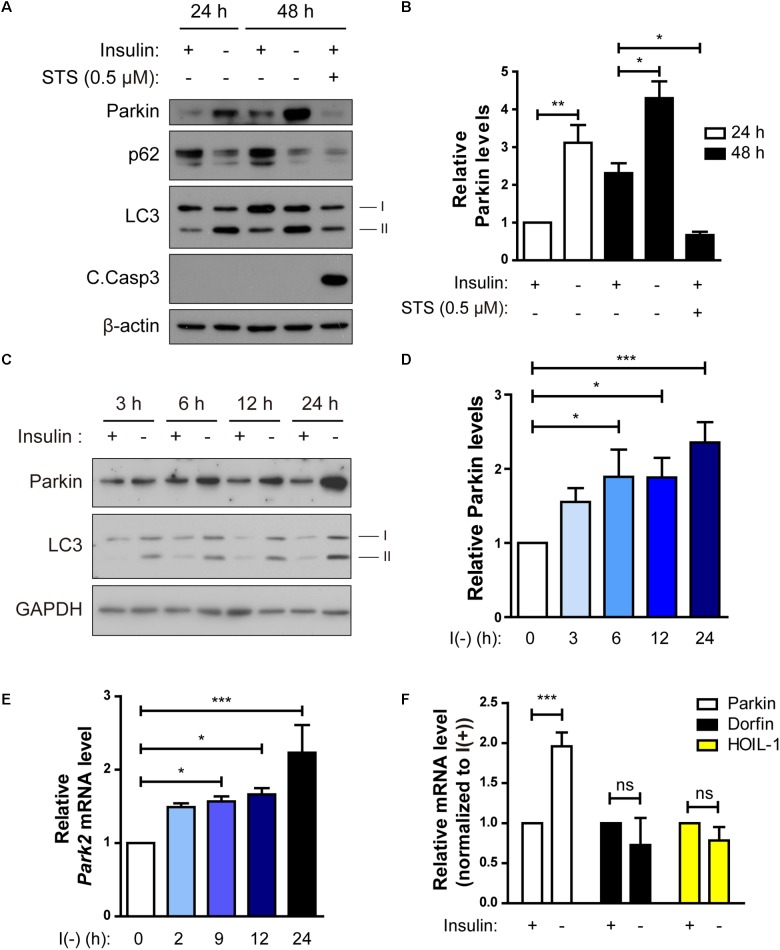
Parkin expression is upregulated following insulin withdrawal. **(A)** Western blotting analysis of Parkin protein level in HCN cells after insulin withdrawal for 24 and 48 h. **(B)** Quantification of Parkin protein level (*n* = 3 or 4). **(C)** Time course analysis of Parkin protein levels following insulin withdrawal. **(D)** Quantification of Parkin protein levels (*n* = 5) from **(C)**. **(E)** Time course analysis of mRNA levels of Parkin following insulin withdrawal (*n* = 4). **(F)** Changes in the mRNA levels of Parkin, Dorfin, and HOIL-1 (*n* = 3–7). ^∗^*p* < 0.05, ^∗∗^*p* < 0.01, ^∗∗∗^*p* < 0.001; ns, not significant.

### Insulin-Deprived HCN Cells Undergo Excessive Mitophagy

Because we observed mitochondrial Ca^2+^ accumulation and depolarization during ADCD, we next examined whether depolarized mitochondria underwent mitophagy. Co-transfection of HCN cells with GFP-LC3 and DsRed-Mito showed that their overlap was increased by insulin withdrawal Figure 3A,B,E). Phosphatidylinositol 3-phosphate (PI3P) is a key signaling lipid for recruitment of autophagy effector proteins to initiate autophagosome generation (Burman and Ktistakis, 2010). DFCP1 contains PI3P-recognizing FYVE domains and binds to PI3P, and thus can serve as a marker of autophagosome nucleation (Axe et al., 2008). Therefore, we assessed translocation of DFCP1 to the vicinity of damaged mitochondria as another measure of mitophagy (Lazarou et al., 2015). I(-) HCN cells showed not only more DFCP1 puncta, but also increased colocalization of DFCP1 and mitochondria in comparison with I(+) HCN cells, confirming the occurrence of mitophagy (Figure 3C,D,F).

As an alternative approach to demonstrate the localization of LC3 in mitochondria, we used subcellular fractionation. Successful separation of mitochondrial and cytosolic fractions was verified by Western blotting analysis using VDAC and β-tubulin as respective markers for these fractions. We observed more LC3-II in the mitochondrial fraction of I(-) HCN cells than in that of I(+) cells (Figure 3G,H). These results suggest that mitophagy is induced in HCN cells following insulin withdrawal.

### Parkin Is Upregulated Through Inhibition of Its Transcriptional Repressor c-Jun in Insulin-Deprived HCN Cells

Parkin-mediated mitophagy has been actively studied (Narendra et al., 2008). However, it remains ill-defined whether Parkin-dependent mitophagy can contribute to cell death. Since we observed mitophagy in insulin-deprived HCN cells undergoing ADCD, we wondered if Parkin is involved in ADCD through regulation of mitophagy. To that end, we first checked Parkin levels in HCN cells following insulin withdrawal. Parkin protein levels were significantly increased 24 and 48 h after insulin withdrawal (Figure 4A,B). We compared the time course of Parkin upregulation with that of autophagy flux induction following insulin withdrawal. An increase in Parkin protein amount was first observed as early as 3 h after insulin withdrawal, which coincided well with an increase in autophagy flux (Figure 4C,D). Related to its protein levels, its transcript levels also increased significantly following insulin withdrawal (Figure 4E). Interestingly, Parkin protein level was decreased when apoptosis was induced by STS, suggesting the potential ADCD-specific role of Parkin in HCN cells (Figure 4A,B). Like Parkin, Dorfin, and HOIL-1 are also RING-IBR-RING-finger domain E3 ligases present in the brain (Niwa et al., 2002; Tanaka et al., 2004). Interestingly, their mRNA levels were not altered in response to insulin withdrawal (Figure 4F).

**FIGURE 5:**
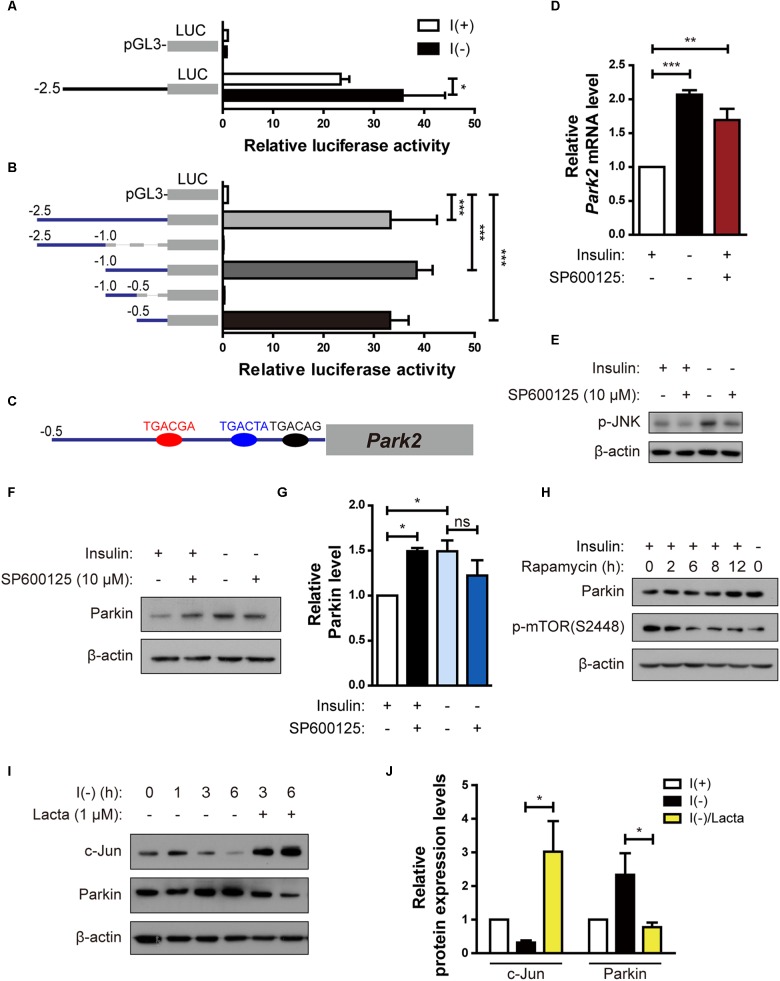
Parkin is upregulated in HCN cells by degradation of c-Jun, a transcriptional repressor, following insulin withdrawal. **(A)** Luciferase activity assay to analyze *Park2* promoter activity after insulin withdrawal for 24 h (*n* = 3). **(B)** Activities of various truncated fragments of the *Park2* promoter in I(+) cells. Promoter activity was measured using a luciferase assay (*n* = 3–7). **(C)** Prediction of c-Jun-binding sites in the *Park2* promoter region. **(D)** Parkin mRNA level in HCN cells treated with SP600125, an ATP-dependent JNK inhibitor, (10 μM) for 8 h in comparison with that in I(-) HCN cells after insulin withdrawal for 24 h (*n* = 3). **(E)** Western blotting analysis of p-JNK levels in HCN cells after insulin withdrawal for 2 h. SP600125 (10 μM) was treated 10 μM for 2 h. The blots shown are representative of three experiments with similar results. **(F)** Western blotting analysis of Parkin protein levels in SP600125 (10 μM, 8 h) -treated HCN cells. The blots shown are representative of five experiments with similar results. **(G)** Quantification of Parkin levels (*n* = 5) from **(F)**. **(H)** Increased Parkin protein levels in HCN cells treated with Rapamycin (20 nM, 2 h), a mTOR inhibitor. (**I**) Time course analysis of c-Jun protein levels following insulin withdrawal in comparison with lactacystin treatment (Lacta, 1 μM). **(J)** Quantification of c-Jun and Parkin protein levels in I(-) HCN cells treated with or without Lacta (1 μM) for 6 h (*n* = 3). ^∗^*p* < 0.05, ^∗∗^*p* < 0.01, ^∗∗∗^*p* < 0.001.

Because upregulation of the Parkin mRNA level by insulin withdrawal seemed to play an important role in ADCD of HCN cells, we investigated the regulation of Parkin gene transcription in insulin-deprived HCN cells. First, to delineate the *Park2* promoter region critical for transcription, we cloned a 2.5-kilobase rat *Park2* promoter fragment in the pGL3-luc vector in front of a sequence encoding luciferase and measured *Park2* promoter activity by luciferase assay. Luciferase expression driven by the cloned *Park2* promoter fragment led to substantial luciferase activity in the basal condition (presence of insulin) in comparison with the empty vector (Figure 5A). Activity was further increased by insulin withdrawal (Figure 5A). Next, we cloned a series of truncated versions of the *Park2* promoter to find the sequences critical for *Park2* transcription and found that 500 bp upstream of the *Park2* gene is the core promoter region (Figure 5B).

Next, we used the PROMO program (Messeguer et al., 2002; Farré et al., 2003) to predict the candidate transcription factors that may participate in both Parkin transcription and autophagy. Binding element analyses predicted several candidate transcription factors, including c-Jun because there were three potential c-Jun-binding sites in the *Park2* promoter region (Figure 5C). c-Jun is a transcription factor phosphorylated and activated mainly by c-Jun NH2 terminal kinase (JNK) (Minden et al., 1994). Based on prior reports on c-Jun functions in autophagy inhibition (Yogev and Shaulian, 2010) and *Park2* transcriptional repression (Bouman et al., 2011), we hypothesized that c-Jun decreases Parkin level. To test this hypothesis, we used an ATP-competitive JNK inhibitor, SP60015, to block phosphorylation of c-Jun by JNK. SP600125 treatment increased Parkin mRNA and protein levels in the presence of insulin, suggesting that *Park2* is under transcriptional repression by c-Jun in the I(+) condition when Parkin level is low (Figure 5D–G). However, Parkin expression levels was not further increased by SP600125 treatment in insulin-deprived HCN cells (Figure 5F,G), probably due to already high Parkin protein level in I(-) HCN cells. Inactivation of the mammalian target of rapamycin (mTOR)-Akt pathway in I(-) HCN cells was observed previously (Yu et al., 2008). To test whether the mTOR-Akt cascade is involved in Parkin expression, we checked the Parkin expression levels in I(+) condition with rapamycin, which is an mTOR inhibitor. We observed increased Parkin expression in rapamycin-treated I(+) HCN cells, suggesting the involvement of mTOR-Akt pathway in regulation of Parkin following insulin withdrawal in HCN cells (Figure 5H).

If c-Jun is a transcriptional repressor of the *Park2* gene, then its level should decrease following insulin withdrawal to allow the upregulation of the Parkin mRNA level. To validate this notion, we assessed changes in c-Jun protein in the absence of insulin. c-Jun protein amount was reduced in a time-dependent manner with kinetics similar to that of Parkin increase (Figure 5I). This gradual decrease in c-Jun level was due to its degradation via the ubiquitin–proteasome system, since the proteasome inhibitor lactacystin prevented c-Jun degradation (Figure 5I,J). Lactacystin-mediated blockage of c-Jun degradation decreased the Parkin protein level under insulin withdrawal conditions, confirming the inhibitory role of c-Jun in Parkin expression (Figure 5I,J). Taken together, these data reveal that c-Jun is a transcriptional repressor of the *Park2* gene, and proteasome-dependent degradation of c-Jun leads to upregulation of Parkin in insulin-deprived HCN cells.

### Parkin Knockout Prevents ADCD in HCN Cells Following Insulin Withdrawal

To investigate the role of Parkin in ADCD of insulin-deprived HCN cells, we over-expressed Parkin, however, there was no significant change in ADCD rate (data not shown). We speculated that Parkin level was already high in insulin-deprived HCN cells and therefor over-expression of Parkin did not induce additional effects. Therefore, we ablated the *Park2* gene in HCN cells by using the CRISPR/Cas9 gene-editing technique and designated the knockout (KO) cells as sgParkin and control cells as sgCon.

**FIGURE 6:**
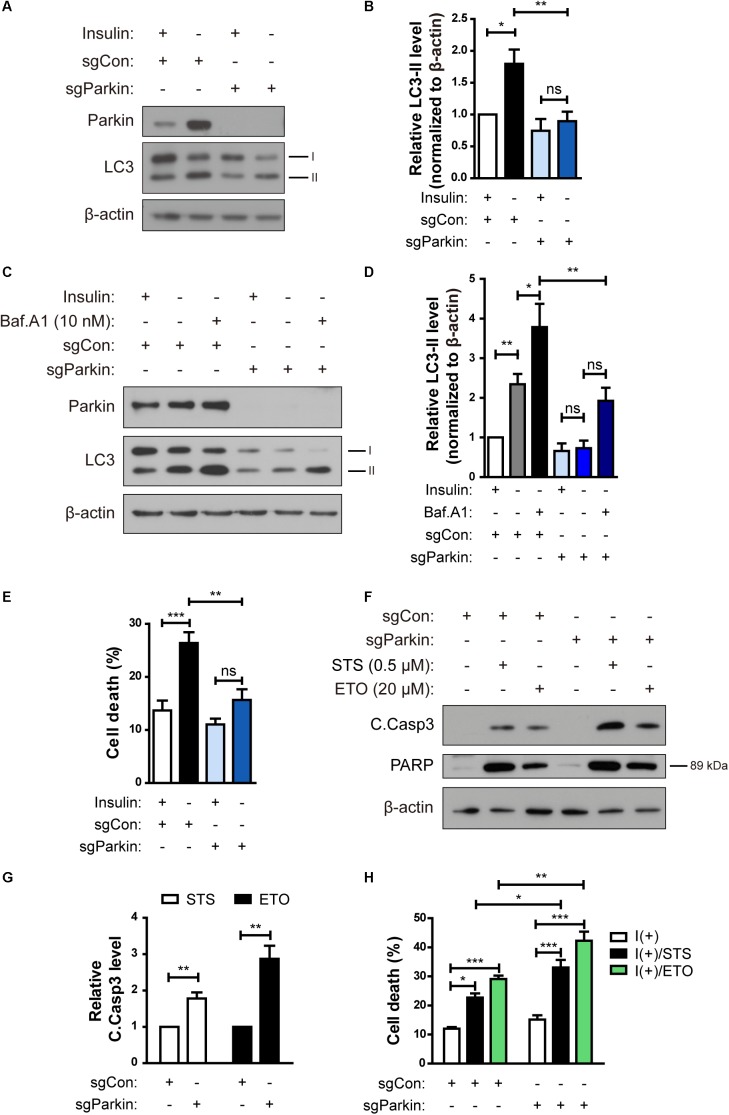
Parkin knockout (KO) prevents ADCD in HCN cells following insulin withdrawal. **(A)** A decrease in autophagy level in sgParkin cells after insulin withdrawal for 24 h. **(B)** Quantification of LC3-II levels (*n* = 6). **(C)** Autophagic flux in sgParkin cells after insulin withdrawal. Cells were treated with Baf.A1 (10 nM) before harvesting. The blots shown are representative of four experiments with similar results. **(D)** Quantification of LC3-II levels (*n* = 4) from **(C)**. **(E)** Cell death rates in sgCon and sgParkin cells after insulin withdrawal for 24 h (*n* = 11). **(F)** Western blotting analysis of C.Casp3, PARP after STS (0.5 μM) or etoposide (ETO) (20 μM) treatment for 8 h. **(G)** Quantification of C.Casp3 level after normalization to β-actin (*n* = 5–7). **(H)** Cell death rates of sgCon and sgParkin cells after STS or ETO treatment for 8 h (*n* = 3). ^∗^*p* < 0.05, ^∗∗^*p* < 0.01, ^∗∗∗^*p* < 0.001.

Parkin KO attenuated an increase in LC3-II levels following insulin withdrawal (Figure 6A,B). Autophagy flux was also decreased in sgParkin cells, as revealed by a decrease in accumulation of LC3-II induced by Baf.A1 (Figure 6C,D). Also, ablation of the *Park2* gene reduced cell death in I(-) HCN cells (Figure 6E). However, Parkin KO did not prevent, but rather promoted apoptosis, since the two prototypical apoptotic inducers STS and etoposide (ETO) increased cell death rate to a greater extent and led to a more robust activation of caspase 3 and PARP in sgParkin than in sgCon cells (Figure 6F–H). These results suggest that Parkin has opposite pro-death or anti-death roles depending on HCN cell death mode. Parkin is essential for insulin withdrawal-triggered mitophagy and plays a pro-death role in ADCD. However, Parkin has anti-apoptotic activity against well-known apoptotic inducers and its deletion renders HCN cells more susceptible to apoptosis.

**FIGURE 7:**
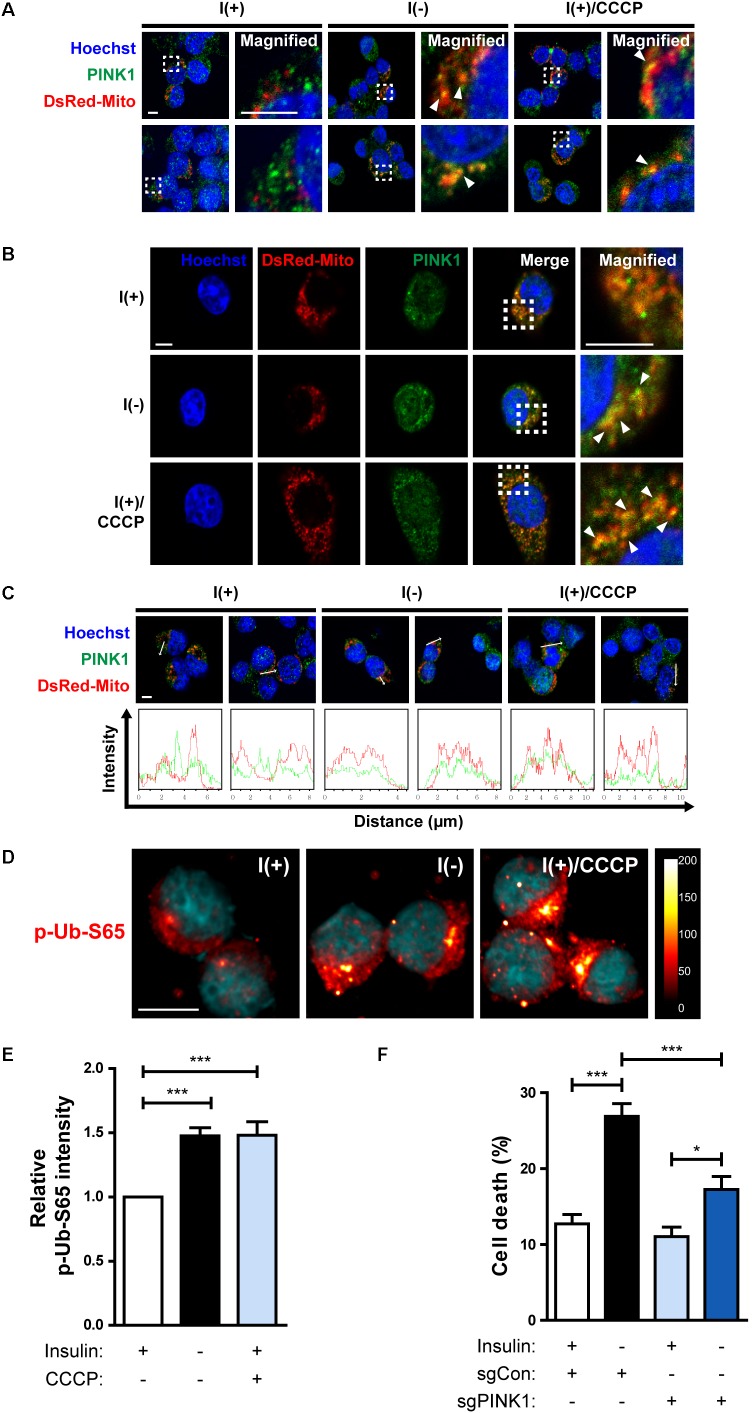
HCN cells undergo PINK1/Parkin-dependent mitophagy following insulin withdrawal. **(A,B)** An increase in colocalization of PINK1 and mitochondria after insulin withdrawal for 24 h. Arrowheads indicate DsRed-Mito and PINK1 colocalization. Scale bars, 5 μm. **(C)** Intensity profile graphs of PINK1 and DsRed-Mito. Scale bar, 5 μm. **(D)** An increase in the level of p-Ub-S65 after insulin withdrawal for 24 h. Cells were treated with CCCP (10 μM) for 0.5 h as a positive control of mitophagy. Blue staining indicates Hoechst, and red staining indicates p-Ub-S65. Intensity is indicated in arbitrary units. Scale bar, 10 μm. **(E)** The mean intensity of p-Ub-S65 was quantified using ImageJ software (*n* = 33 cells for I(+), 38 cells for I(−), 46 cells for I(+)/CCCP from 3 independent experiments). **(F)** Cell death rates of sgCon and sgPINK1 cells (*n* = 4). ^∗^*p* < 0.05, ^∗∗∗^*p* < 0.001.

In the Parkin-dependent mitophagy model, PINK1 is essential for the action of Parkin (Narendra et al., 2010; Youle and Narendra, 2011). PINK1 constitutively shuttles between the cytosol and mitochondria in normal cells but accumulates on the outer membrane of depolarized mitochondria (Narendra et al., 2010). Therefore, we examined whether PINK1 is recruited to mitochondria in I(-) HCN cells. Fluorescence imaging analyses showed an increased accumulation of PINK1 on the mitochondria in I(-) HCN cells (Figure 7A–C). To further examine the involvement of PINK1 in mitophagy, we used an anti-p-Ub-S65 antibody. PINK1 is a ubiquitin kinase. Once stabilized on the outer membrane the depolarized mitochondria, PINK1 phosphorylates ubiquitin at S65, which further stimulates the E3 ligase activity of Parkin (Kane et al., 2014). As expected, in accordance with PINK1 accumulation, the signal intensity p-Ub-S65 was substantially increased in I(-) HCN cells (Figure 7D,E). In this experiment, the potent depolarizing agent CCCP was used as a positive control to chemically induce mitophagy (Figure 7A–D). In addition, ADCD was reduced by PINK1 KO (Figure 7F). In conclusion, PINK1 acts upstream of Parkin in insulin-deprived HCN cells, and HCN cells undergo ADCD through PINK1/Parkin-dependent mitophagy.

### Parkin KO Rescues Mitochondrial Alterations and Prevents Initiation of Mitophagy in Insulin-Deprived HCN Cells

**FIGURE 8:**
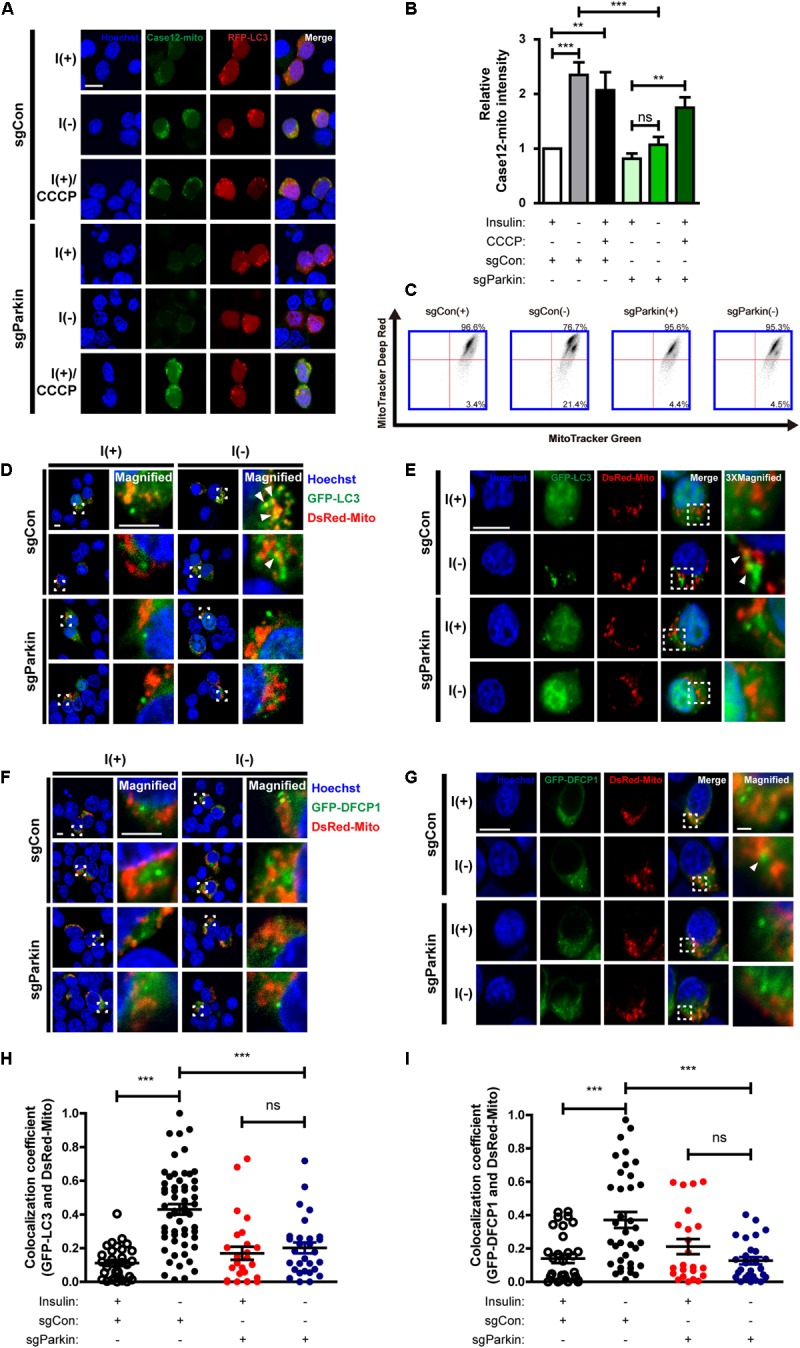
Parkin KO prevents initiation of mitophagy following insulin withdrawal. **(A)** Fluorescence images of Case12-mito in sgParkin cells after insulin withdrawal for 24 h. Scale bar, 5 μm. **(B)** Quantification of fluorescence intensity of Case12-mito (*n* = 39 cells for sgCon(+), 28 cells for sgCon(-), 36 cells for sgCon(+)/CCCP, 80 cells for sgParkin(+), 73 cells for sgParkin(-), 37 cells for sgParkin(+)/CCCP from 3 independent experiments). **(C)** Double staining with MitoTracker Green and MitoTracker Deep Red to measure the ratio of depolarized mitochondria relative to total mitochondria after insulin withdrawal for 24 h. **(D,E)** Colocalization of LC3 and mitochondria in sgParkin cells after co-transfection with GFP-LC3 and DsRed-Mito. Colocalization was analyzed after insulin withdrawal for 24 h. Arrowheads indicate colocalization of GFP-LC3 and DsRed-Mito. Scale bars, 5 μm for **(D)**, 10 μm for **(E)**. **(F,G)** Colocalization of DFCP1 and mitochondria (arrowheads) in sgParkin cells after insulin withdrawal for 24 h. Scale bars, 5 μm for **(F)**, 10 μm for **(G)**. **(H)** Quantification of colocalization of LC3 and mitochondria (*n* = 26–52 cells from 3 independent experiments). **(I)** Quantitative analysis of colocalization of DFCP1 and mitochondria (*n* = 23–36 cells from 3 independent experiments). ^∗∗^*p* < 0.01, ^∗∗∗^*p* < 0.001; ns, not significant.

Next, we examined whether Parkin deletion affects events upstream of mitophagy. Interestingly, mitochondrial Ca^2+^ level was reduced in sgParkin cells (Figure 8A,B). Unexpectedly, we found that the ratio of depolarized mitochondria was lower in sgParkin than sgCon cells following insulin withdrawal (Figure 8C). These results suggest that Parkin is required for efficient ER-to-mitochondria Ca^2+^ transfer and ensuing depolarization. In accordance with decreases in the mitochondrial Ca^2+^ accumulation and depolarization, subsequent mitophagy-related events, such as mitochondrial translocation of LC3 (Figure 8D,E,H) and DFCP1 (Figure 8F,G,I), were all attenuated in I(-) sgParkin cells, suggesting that Parkin KO interfered with the initial steps of mitophagy. Our results suggest that Parkin deletion not only impairs the recognition of the depolarized mitochondria and their removal, but also blocks early changes in mitochondrial physiology and prevents initiation of mitophagy. Therefore, mechanistically, Parkin is intimately involved in mitophagy from its very early steps, including ER-to-mitochondria Ca^2+^ transfer and recruitment of the autophagy-initiating/elongating molecules to mitochondria.

**FIGURE 9:**
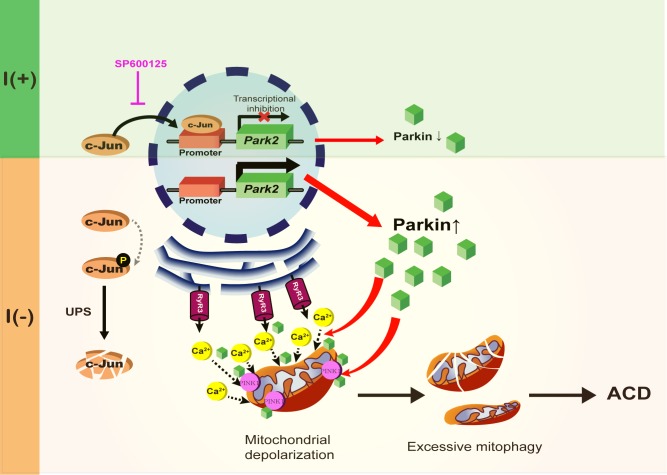
A schematic diagram of Parkin-dependent mitophagy and ADCD. Parkin expression is increased by the degradation of c-Jun, a transcriptional repressor of Parkin in insulin-deprived HCN cells. Insulin withdrawal induced mitochondrial depolarization and recruitment of Parkin and PINK1 to mitochondria, where Parkin is also required for ER-to-mitochondria Ca^2+^ transfer. PINK1/Parkin-dependent mitophagy occurs in HCN cells following insulin withdrawal causing excessive mitophagy and ADCD.

## Discussion

Parkin can suppress or promote apoptosis depending on cell type and stressor. Our results are more in line with the anti-apoptotic role of Parkin in HCN cells challenged with well-known apoptotic stimuli. In contrast to this pro-survival role, we also found that Parkin promotes ADCD following the removal of insulin, the key survival neurotrophic factor for HCN cells. Currently, it is not known how these opposite roles of Parkin in the control of distinct modes of cell death are regulated. Overall, deregulated Parkin activity and mishandling of depolarized mitochondria will be detrimental to the cells and lead to neurodegeneration through various pathways depending on cell type and degeneration cue.

Although, PD is mainly a movement disorder with motor symptoms, non-motor symptoms such as sleep abnormalities, autonomic failure, and a range of neuropsychiatric symptoms including depression, anxiety, cognitive impairment, dementia, and impulse control disorders are increasingly being recognized as features of PD pathology (Meissner et al., 2011). So far, most therapeutic efforts have been focused on motor control, and alleviation of non-motor symptoms has received less attention. Therefore, there is an unmet need to alleviate non-motor symptoms for better therapeutic design and management of PD. However, currently there is no good animal or cellular model to help address the neuropsychiatric symptoms of PD. In that regard, our present study on Parkin-mediated mitophagy in HCN cells could contribute to understanding the various roles of Parkin in brain areas other than substantia nigra.

What is the interconnection between mitophagy and conventional autophagy? Although, it may not be easy to distinguish the effects of mitophagy from those of conventional bulk autophagy in the same cell, it is interesting that ablation of Parkin attenuates not only mitophagy, but also conventional autophagy. Parkin may play additional roles in facilitation of conventional autophagy in insulin-deprived HCN cells. Another possibility is that elimination of mitochondria has a greater effect on autophagy than elimination of other intracellular constituents in HCN cells. Since interference with mitophagy by using Mdivi-1 reduced autophagy level in HCN cells, the latter possibility seems plausible. During bulk autophagy, as in nutrient starvation conditions, autophagy can be selective for certain intracellular organelles, such as ribosomes and peroxisomes (Hara-Kuge and Fujiki, 2008; Cebollero et al., 2012). In certain cell types, including HCN cells, mitochondria could be the main cargo for autophagic degradation.

In this study, we also identified a new mode of action of Parkin in regulation of mitophagy. Our data suggest that ER-to-mitochondria Ca^2+^ mobilization triggers Parkin/PINK1-dependent mitophagy and at the same time Parkin is required for this Ca^2+^ mobilization. In line with this idea, a previous study reported that Parkin upregulation increases ER? mitochondria contact to regulate Ca^2+^ transfer (Calì et al., 2013). Upon insulin withdrawal, Parkin mediates Ca^2+^ accumulation in mitochondria and thereby instigates mitochondrial depolarization at the early stage of mitophagy. This may allow recruitment of PINK1 and Parkin and progress of mitophagy. Then, recruited Parkin recognizes and removes dysfunctional mitochondria. According to our model, Parkin is intimately involved from the beginning of mitophagy, and liaises with PINK1 in a mutually cooperative way for the progress of mitophagy. Parkin’s role is more than recognition and removal of depolarized mitochondria; it is an initiator and actuator of mitophagy in the context of ADCD. A schematic diagram shown in Figure 9 summarizes the regulation of Parkin expression and its roles during ADCD.

It is also puzzling how Parkin-mediated selective elimination of dysfunctional mitochondria leads to cell death in insulin-deprived HCN cells, whereas Parkin-dependent mitophagy promotes and cellular homeostasis according to other studies. Currently, the link between mitophagy and cell death remains under-studied. What mechanisms determine the outcome of Parkin-driven mitophagy, i.e., whether it ensures mitochondrial quality control or facilitates mitophagic cell death? Further studies are warranted to resolve this issue.

## Author Contributions

S-WY contributed to conception and design, data analysis and interpretation, manuscript writing, and approved the manuscript. HP contributed to conception and design, collection and assembly of data, data analysis and interpretation, and manuscript writing. KMC and H-KA contributed to data collection and analysis. BC and CM contributed to study design, and data analysis and interpretation. J-EG, JH, and HW collected the data.

## Conflict of Interest Statement

The authors declare that the research was conducted in the absence of any commercial or financial relationships that could be construed as a potential conflict of interest.
